# Superior effectiveness and acceptability of saliva samples for the detection of SARS-CoV-2 in China

**DOI:** 10.1016/j.bsheal.2024.03.002

**Published:** 2024-03-05

**Authors:** Hui Yao, Ying Shen, Zhichao Liang, Xiaoyu Xue, Chenxi Zhao, Xiang Xu, Yuxin Cai, Yonghong Liu, Wei Zhang, Yang Pan, Xiaoli Wang

**Affiliations:** a Beijing Office of Global Health, Beijing Center for Disease Prevention and Control, Beijing 100013, China; b Institute for Infectious Disease and Endemic Disease Control, Beijing Center for Disease Prevention and Control, Beijing 100013, China; c National Key Laboratory of Intelligent Tracking and Forecasting for Infectious Diseases, Beijing Ditan Hospital, Capital Medical University, Beijing 100015, China; d Center for Drug Evaluation, National Medical Products Administration, Beijing 100176, China; e School of Public Health, Capital Medical University, Beijing 100069, China

**Keywords:** SARS-CoV-2, Saliva sample, Sensitivity, Acceptability

## Abstract

In response to problems of poor sampling quality, low sensitivity, and high demand for medical personnel regarding the current severe acute respiratory syndrome coronavirus 2 (SARS-CoV-2) oropharyngeal (OP) swab sampling used in China, we aimed to evaluate the diagnostic performance and acceptability of saliva-based nucleic acid amplification tests (NAATs) in China. The results showed that, using nasopharyngeal (NP) swab results as the gold standard, the overall sensitivities for saliva specimens and OP swabs were 93.3 % and 85.0 %, the specificities were 92.6 % and 93.8 %, respectively. The results of an acceptability survey showed that the scores for saliva, OP, and NP samples were 9.46 ± 1.69, 8.11 ± 2.42, and 4.58 ± 3.82 out of 10, respectively, with significant differences among the three groups (*P* < 0.05). With higher sensitivity, comparable specificity, and strong public preference, saliva-based NAATs represent a convenient and effective method for detecting SARS-CoV-2 in future epidemics.

## Highlights

• Effectiveness and acceptability of saliva-based nucleic acid amplification tests (NAATs) for detecting severe acute respiratory syndrome coronavirus 2 (SARS-CoV-2) was evaluated.
• Using nasopharyngeal (NP) swab results as the gold standard, the sensitivities for saliva and oropharyngeal (OP) swabs were 93.3 % and 85.0 %; specificities were 92.6 % and 93.8 %, respectively.
• The acceptability scores for saliva, OP, and NP swabs were 9.46 ± 1.69, 8.11 ± 2.42, and 4.58 ± 3.82 out of 10, respectively.
• Saliva-based NAATs might be a convenient and effective method for detecting SARS-CoV-2 in future epidemics.

## 1 Introduction

Many countries have implemented saliva-based nucleic acid amplification tests (NAATs) for detecting severe acute respiratory syndrome coronavirus 2 (SARS-CoV-2), which have demonstrated satisfactory diagnostic performance [1], [2], [3] and acceptability [4], [5] for case identification. A comprehensive review thoroughly examined the successful implementation of saliva-based SARS-CoV-2 testing worldwide, highlighting its excellent performance in detection and its broad acceptance [6]. In response to the problems of poor sampling quality, low sensitivity, and high demand for medical personnel in the current SARS-CoV-2 oropharyngeal (OP) swab sampling used in China [7], in this study, we aimed to evaluate the diagnostic performance of NAAT outcomes derived from saliva specimens within the Chinese population. Additionally, we investigated the acceptability and preferences among the public of saliva specimens, OP swabs, and nasopharyngeal (NP) swabs as sampling methods.

## 2 Materials and methods

Study recruitment was conducted from February 2023 to April 2023. A total of 656 participants were recruited, with 124 participants from the community and 532 hospital outpatients or inpatients; participants were sampled by the Beijing Centers for Disease Control (Beijing CDC) and the corresponding hospitals, respectively. All collected specimens were tested by the Beijing CDC using quantitative reverse transcription polymerase chain reaction (qRT-PCR). NP swab, OP swab, and saliva specimens were collected at the same time after participant enrollment, and cycle threshold (Ct) values of the NAAT results were tested for all samples. Participants with negative test results were defined as those with an NP Ct value ≥40 cycles [8]*.* Relevant information was collected for all participants, including their identification number, age, sex, occupation, sample type, and test results.

An online questionnaire survey was administered to 156 sampled participants to investigate the acceptability of sampling using NP swabs, OP swabs, and saliva and their preference, ranked among the four sampling methods, the questionnaire details are available in Table S1. Participants rated their acceptability of NP swab, OP swab, and saliva specimen sampling on a 10-point scale, from very unwilling to accept (0) to very willing to accept (10). The four sampling strategies included “having OP swabs collected by a professional at a designated location” “collecting a saliva specimen at home and then sending it to a designated location”, “self-collected nasal swabs for rapid antigen testing at home”, and “having NP swabs collected by a professional at a designated location”.

All statistical analyses were performed using R version 4.2.1 and RStudio. Test results for saliva and OP specimens were decided using NP results as the gold standard and presented with calculation of the sensitivity and specificity. We calculated 95 % confidence intervals (CIs) with the Wilson score CI, and consistency of the test results was estimated using the kappa value. The receiver operating characteristic (ROC) and area under the curve (AUC) values for saliva and OP specimens were calculated, and the Delong test was used to compare the difference between AUC values. The questionnaire was analyzed to assess the acceptability of each sample type, with the mean score and standard deviation (SD) utilized as descriptive statistics. A *P*-value of < 0.05 was deemed statistically significant.

## 3 Results

We collected a total of 1,968 samples from 656 participants, including 656 NP swabs, 656 OP swabs, and 656 saliva specimens. Of the 656 participants, 282 (42.9 %) were male. The median participant age was 31 years old (interquartile range: 22 - 48), with the youngest aged 14 years and the oldest aged 93 years. Of the 607 participants with occupation and workplace information, 268 (44.2 %) were school teachers or students, 157 (25.9 %) were retired or unemployed, 90 (14.8 %) were office workers, and 92 (15.1 %) worked in various kinds of service sectors such as in hospitals, business services, and catering services.

**Fig. 1:**
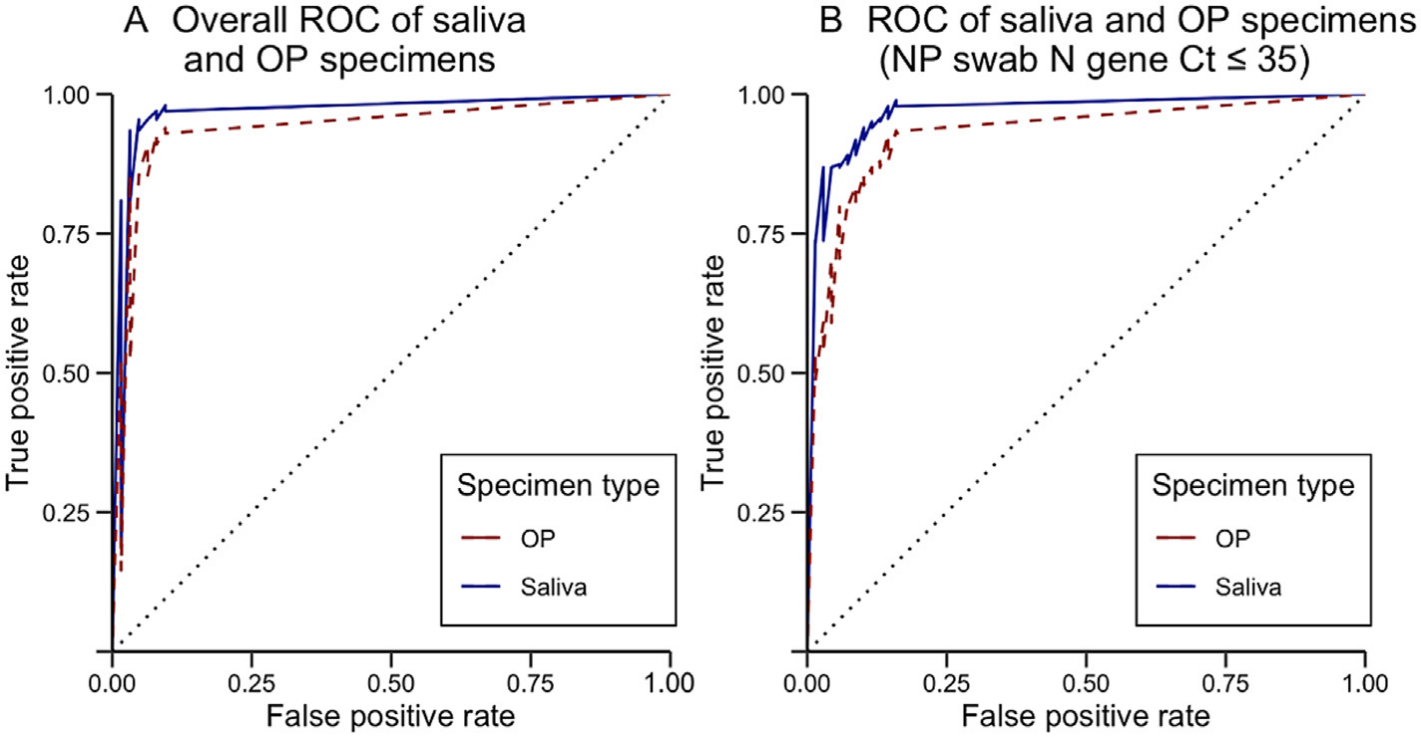
ROC of saliva and oropharyngeal specimens. A) Overall ROC of saliva and oropharyngeal specimens. B) ROC of saliva and oropharyngeal specimens (NP swab Ct values < 35). Abbreviations: ROC, receiver operating characteristic; OP, oropharyngeal; NP, nasopharyngeal; Ct, cycle threshold.

Among qRT-PCR results for the 656 NP swabs, 494 (75.3 %) were identified as SARS-CoV-2 positive. Using NP swab results as the gold standard, the overall sensitivities for saliva specimens and OP swabs were 93.3 % (95 % CI: 90.8 % - 95.2 %) and 85.0 % (95 % CI: 81.6 % - 87.9 %), respectively; the overall specificities were 92.6 % (95 % CI: 87.5 % - 95.7 %) and 93.8 % (95 % CI: 89.0 % - 96.6 %), respectively (Table 1). The saliva results showed better consistency with the NP swab results, with a kappa value of 0.823, in comparison with the OP swab results, which had a kappa value of 0.696. The AUC was calculated for both specimens; the AUC for saliva specimen results was 0.971, and the AUC for OP swab results was 0.943 (Fig. 1A). The Delong test showed a statistically significant difference between the two AUC values (*P* < 0.05).

**Table 1:** Sensitivity and specificity of saliva and oropharyngeal swab by different characteristics.

Characteristics		n (%)	Saliva		OP swab	
			Sensitivity (%)	Specificity (%)	Sensitivity (%)	Specificity (%)
Samples with different viral loads a	Ct ≤ 40	653 (99.5)	93.3	93.7	85.0	94.3
	Ct ≤ 35	616 (93.9)	95.1	97.3	86.3	97.3
	Ct ≤ 30	545 (83.1)	97.2	97.3	88.9	97.3
	Ct ≤ 25	404 (61.6)	97.6	97.3	92.5	97.3

Occupation b	Students & teachers	268 (44.2)	91.9	91.4	78.6	94.8
	Retired & unemployed	157 (25.9)	96.2	96.3	91.5	100.0
	Office workers	90 (14.8)	90.9	94.3	80.0	94.3
	Others	92 (15.1)	90.6	92.3	92.5	89.7

Age group (years)	[0, 18)	42 (6.4)	92.9	N/A^c^	85.7	N/A^c^
	[18, 35)	345 (52.6)	93.2	91.6	79.6	93.7
	[35, 60)	186 (28.3)	90.6	98.3	90.6	98.3
	[60, +)	83 (12.7)	98.6	66.7^c^	93.2	66.7^c^

Total		656	93.3	92.6	85.0	93.8

Abbreviations: OP, oropharyngeal; NP, nasopharyngeal; Ct, cycle threshold; N/A, not applicable.

a Using the nasopharyngeal swab N gene Ct value as the cut-off point, samples with corresponding Ct values were included in the calculation of sensitivity and specificity.

b A total of 607 participants with occupation information were included. For this analysis, participants were categorized according to their occupation. The category “others” includes participants who worked in service sectors, such as medical services, business services, catering services, etc.

c The study was unable to recruit enough NP test negative participants under 18 years old or over 60 years old, thus specificity of participants under 18 years of age was not applicable, and the specificity of participants over 60 years of age might not be reliable.

To further explore the diagnostic efficacy in participants with varying viral loads, we computed the sensitivity, specificity, and AUC for both saliva and OP specimens across different Ct intervals, based on the corresponding NP nucleocapsid gene Ct values. Saliva specimens consistently exhibited higher sensitivities and comparable specificities, in comparison with OP swabs, across each Ct interval (Table 1). Among individuals with NP swab Ct values < 35 cycles (n = 616), the AUC for saliva specimens was 0.977 and the AUC for OP swabs was 0.932 (Fig. 1B). The Delong test showed a significant difference between the two AUCs (*P* < 0.05). The diagnostic performance by age group showed that saliva specimens had higher sensitivities than OP specimens in the age groups 0–18 years (92.9 % vs. 85.7 %) and 18–35 years (93.2 % vs. 79.6 %) (*P* = 0.32); the specificities were similar for age groups 18–35 and 35–60 years (*P* = 0.10) (Table 1). To explore whether saliva specimens are more suitable for specific scenarios or settings, sensitivities and specificities for participants with different occupations were assessed. The sensitivity of saliva specimens among students and teachers exhibited better performance in comparison with OP swabs (91.9 % vs. 78.6 %). Satisfactory performance was also exhibited for specimens from participants with other occupations (Table S2). However, both sensitivities and specificities for these two types of specimens showed no statistical significance between groups, with *P*-values of 0.47 and 0.16, respectively.

The survey results revealed that the most favored sampling method among participants was saliva specimen collection, which obtained a mean score 9.46 ± 1.69. This was followed closely by OP swab collection, with a score of 8.11 ± 2.42; the score for NP swabs was lowest, at 4.58 ± 3.82 (Table S2). Notably, there was a statistically significant difference in acceptance levels observed among these three sampling methods (*P* < 0.05). The acceptability score by occupation showed that in a comparison of saliva specimens and NP swabs, retired and unemployed participants (9.74 ± 0.82 vs. 4.40 ± 3.87) and office workers (9.70 ± 0.67 vs. 3.30 ± 3.16) reported significantly higher acceptability of saliva specimen collection over NP swab collection (Table S3).

In terms of the most preferred options among the four sampling strategies, the highest preference was “collecting a saliva specimen at home then delivering it to a designated location,” with 68.0 % ranking this option among their top two choices. Following this, “having OP swabs collected by a trained professional at a specified location” was the second most favored choice, with 60.1 % of respondents ranking it among their top two choices (Table S4). The observed disparities in preference rankings across these four options were found to be statistically significant (*P* < 0.05).

## 4 Discussion

This study provides substantial evidence to support the use of saliva specimens as a highly sensitive and acceptable alternative to OP swabs for SARS-CoV-2 detection in the Chinese population. The main finding is aligned with previous research highlighting the potential of saliva as a non-invasive and easily collectible sample for diagnostic purposes related to SARS-CoV-2 [9]. Implementing saliva-based testing approaches can likely enhance the accessibility and accuracy of testing, thereby expanding the population who would agree to be tested and reducing the burden on medical resources.

The test results demonstrated that NAATs of saliva specimens exhibited higher sensitivity in comparison with OP swabs (93.3 % vs. 85.0 %), suggesting that saliva might be a more reliable specimen for the detection of SARS-CoV-2. The AUC values and Delong test reinforced the finding regarding the superiority of saliva specimens over OP swabs, indicated by the higher AUC for saliva compared with OP swabs (0.971 vs. 0.943, *P* < 0.05). In past epidemic management, challenges have arisen in detecting cases with low viral loads [10]. However, this study demonstrated that saliva consistently outperformed OP swabs across different Ct intervals, especially in cases with higher Ct values (i.e., lower viral loads).

Considering different occupations and age groups, saliva specimens demonstrated equivalent or higher sensitivity as compared with OP swabs. It is worth noting that the sensitivity of OP swabs was relatively low compared with that of saliva specimens for office workers, which might be owing to poor sampling quality because these participants had less willingness to cooperate with OP swab collection (Table S3). This result was consistent with and might mutually explain the finding that participants aged 18 - 35 years showed lower sensitivity for OP swabs compared with saliva specimens (Table 1). Overall, these results suggest that regardless of age, saliva specimens might offer a reliable alternative to OP swabs for detecting SARS-CoV-2. Additionally, saliva specimens can easily be self-collected in office buildings for large-scale screening during potential outbreaks without the need for a large number of medical personnel.

The participant preference survey reaffirmed the practical feasibility of using saliva-based testing strategies. The marked preference for saliva specimens (9.46 ± 1.69), significantly surpassed that of NP swabs (4.58 ± 3.82) and aligned with other research reports that saliva and OP swabs are the preferred sample types [11]. This clear inclination among the public toward saliva sampling suggests its potential as an ideal option in massive screening as more individuals are likely to participate in screening, as reported in other studies [12]. Importantly, the present study also provides valuable insights into optimal sample collection methods. Most participants favored the option of collecting saliva specimens at home and delivering them to a designated location, indicating strong public acceptance of this approach, especially among participants who worked in an office and retired or unemployed participants who rated saliva specimens higher than OP swab collection, using NP as the standard (Table S2). This finding is crucial for informing public health strategies. The use of the above approach in the community or office settings aligns with the goal of minimizing the potential burden on medical personnel during coronavirus disease 2019 (COVID-19) outbreak [13].

## Ethics statement

This study was approved by the Ethics Committee of the Beijing Center for Disease Prevention and Control (No.202225), and written informed consent was obtained from all participants.
