# Supplementary material for: Superior effectiveness and acceptability of saliva samples for the detection of SARS-CoV-2 in China
Source: Biosaf Health. 2024 Mar 5;6(2):88–91. doi: 10.1016/j.bsheal.2024.03.002 (PMC11894993; doi:10.1016/j.bsheal.2024.03.002)
Supplement: Supplementary data 1 [file mmc1.docx]

**Supplementary material:**

**Method**

*Specimen collection and preservation*

Nasopharyngeal (NP) swab / Oropharyngeal (OP) swab collection: The OP/NP swab was collected by trained personal following a uniform sampling method. The specimens were preserved in sampling tube with 2-3 mL virus-preserving medium in the tube.

Saliva specimen collection: saliva was collected by spitting 2 mL of saliva into a clean container tube. Then 3ml transport and preservation medium was added to the specimen tube.

Specimen preservation: All specimens were stored at 4 °C for no more than 24 hours. If timely testing is not possible, specimens were preserved below -20 °C for a tentative period of no more than 2 months, and repeated freezing and thawing were avoided. All specimens were stored at -80 °C for long-term storage for subsequent use.

*SARS-CoV-2 detection: qRT-PCR assay*

For the extraction of nucleic acid in specimen, 200 uL specimen of saliva, oropharyngeal swabs and nasopharyngeal swabs were taken, and nucleic acid extraction was performed using a Tianlong automated nucleic acid extractor (GeneRotex 96, Xi'an Tianlong Technology Co., Ltd., Xi'an, China). Nucleic acid extraction reagents used were from Xi'an Tianlong Technology Co. Ltd (CqEx-DNA/RNA virus (CDC), Xi'an, China). The test assay used for quantitative reverse transcription polymerase chain reaction (qRT-PCR) was from Guangzhou Daan Genetics Co. (Detection Kit for 2019-nCoV (PCR-Fluorescence), DA0992, Guangzhou, China). Testing instrument used was QuantStudio™ 7 Flex Real-time fluorescence PCR detector (Applied Biosystems by Thermo Fisher Scientific, USA). The dye setting channels were FAM, VIC, and Cy5. The results were automatically saved after the reaction, and the analysis results were automatically obtained by adjusting the start value, end value, and threshold value of baseline according to the analyzed image. For quality control, 2019-nCoV negative quality control must meet requirements below: no Ct value or no obvious amplification curve for FAM and VIC detection channels, Cy5 ≤ 25; 2019-nCoV positive quality control must meet requirements below: Ct value ≤ 22 for FAM and VIC detection channels; the above requirements must be met in the same experiment, otherwise the experiment is invalid and must be repeated. According to the test results of clinical samples, the ROC curve method is used to finally determine that the positive judgment value of the target genes N and ORF1ab of this kit is 40, and the positive judgment value of the internal standard gene RnaseP is 40.

Table S1. Questionnaire setting

| Questions | Measurements |
| --- | --- |
| 1. Your name: |  |
| 1. How receptive are you when the following specimens are collected (0 points means unwilling to accept, 10 points means very willing to accept)? | Score scale |
| - Nasopharyngeal swab | 0-10 |
| - Oropharyngeal swab | 0-10 |
| - Saliva | 0-10 |
| 1. If you can choose sampling strategies in the future, please rank the following strategies according to your own feelings and preferences (the first one is your favorite method). | Ranking |
| - Having oropharyngeal swab collected by a professional at a designated location | 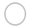1 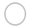2 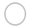3 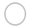4 |
| - Collecting saliva specimen at home and send it to a designated location | 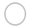1 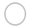2 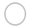3 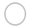4 |
| - Self-collected nasal swab for rapid antigen test at home | 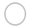1 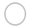2 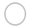3 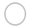4 |
| - Having nasopharyngeal swab collected by a professional at a designated location | 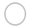1 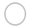2 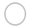3 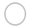4 |

Table S2. Acceptability score of different type of specimens

| Sample type / Score ^a^ | 0-3, n (%) | 4-6, n (%) | 7-10, n (%) | Score (Mean ± SD) |
| --- | --- | --- | --- | --- |
| Nasopharyngeal swab | 72  (46.2) | 32  (20.5) | 52  (33.3) | 4.58 ± 3.82 |
| Oropharyngeal swab | 9  (5.8) | 26  (16.7) | 121  (77.6) | 8.11 ± 2.42 |
| Saliva | 5  (3.2) | 1  (0.6) | 150  (96.2) | 9.46 ± 1.69 |

^a^ Score ranges from 0 to 10, 0 means do not prefer the type of sample. This table showed the count and the percentage of the score given by sampled participants.

Table S3. Acceptability score of different types of specimens by occupation

| Occupation  /Score | n | NP swab  (Mean ± SD) | OP swab  (Mean ± SD) | Saliva  (Mean ± SD) | OP swab vs. NP swab ^a^  (%) | Saliva vs.  NP swab ^a^  (%) |
| --- | --- | --- | --- | --- | --- | --- |
| Retired &  unemployed | 43 | 4.40 ± 3.87 | 8.28 ± 1.91 | 9.74 ± 0.82 | 88.2 | 121.4 |
| Students &  teachers | 36 | 4.97 ± 3.57 | 8.17 ± 2.43 | 8.81 ± 2.41 | 64.4 | 77.3 |
| Office workers | 10 | 3.30 ± 3.16 | 6.30 ± 3.06 | 9.70 ± 0.67 | 90.9 | 193.9 |
| Others | 17 | 5.29 ± 4.45 | 8.12 ± 2.74 | 9.35 ± 2.42 | 53.5 | 76.7 |

^a^ The increase in acceptability score by occupation was calculated for different samples using the corresponding NP swab scores as the standards.

Abbreviations: OP, oropharyngeal; NP, nasopharyngeal.

Table S4. Ranking of different sample strategies

| Sample strategy options/Rank | n | No.1, n (%) | No.2, n (%) | No.3, n (%) | No.4, n (%) |
| --- | --- | --- | --- | --- | --- |
| Having oropharyngeal swab collected by a professional at a designated location | 153 | 53 (34.6) | 39 (25.5) | 50 (32.7) | 11 (7.2) |
| Collecting saliva specimen at home and send it to a designated location | 147 | 52 (35.4) | 48 (32.6) | 31 (21.1) | 16 (10.9) |
| Self-collected nasal swab for rapid antigen test at home | 145 | 36 (24.8) | 30 (20.7) | 38 (26.2) | 41 (28.3) |
| Having nasopharyngeal swab collected by a professional at a designated location | 147 | 15 (10.2) | 32 (21.8) | 25 (17.0) | 75 (51.0) |
